# Indicator rating methodology for Rapid Sustainability Assessment Method (RSAM) for existing residential buildings using opinions of residents

**DOI:** 10.1016/j.mex.2020.101105

**Published:** 2020-10-16

**Authors:** Ferhat Karaca, Mert Guney, Aiganym Kumisbek

**Affiliations:** a Environmental Science & Technology Group (ESTg), Department of Civil and Environmental Engineering, Nazarbayev University, 010000 Nur-Sultan, Kazakhstan; b The Environment and Resource Efficiency Cluster (EREC), Nazarbayev University, 010000 Nur-Sultan, Kazakhstan

**Keywords:** Green building, Householder opinion, Kazakhstan, Sustainability assessment tools, Sustainability ranking, Sustainability rating

## Abstract

The presented method provides the details for how each indicator in “RSAM: A Rapid Sustainability Assessment Method” is scored using a residents’ opinions-based sustainability assessment procedure that is specifically designed for the assessment of existing residential buildings. Existing methods in the literature mostly suggest indicator scores in construction sustainability assessments using highly technical data and require a high level of engineering and management expertise. This makes them elaborate but labor-, time-, and cost-intensive; and thus, their use is more frequently favored in new projects.

• The presented method redefines a set of conventional environmental, social-functional, and economic indicators in a context which combines data from residents’ opinions (e.g. perceived quality of indoor temperature comfort level) and easy-to-obtain information (e.g. annual utility bills).
• All indicator ratings are represented within a 10-point scale, and their weights on the overall model are identified by stakeholders.
• RSAM provides a rapid and low-cost assessment for existing buildings which normally may not go through a conventional sustainability assessment due to resource limitations.

## Graphical abstract

**Figure:**
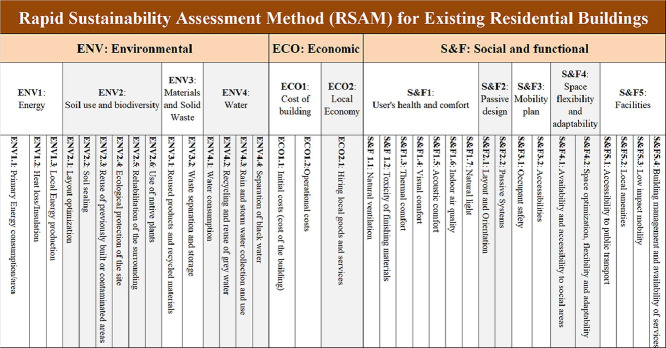

Specifications table

**Table:** 

Subject Area	Environmental Science
More specific subject area	*Building sustainability*
Method name	*RSAM (Rapid Sustainability Assessment Method)*
Name and reference of original method	*none*
Resource availability	*Not applicable*

## *Method details

The RSAM method was developed by performing a detailed analysis of the current building requirements/concepts. All of the selected sustainability assessment factors and indicators are hierarchically subcategorized under factors, then indicators, and finally sub-indicators with regard to their relevance to each other. They were carefully selected from the existing literature to measure residents’ opinions on contemporary applications of building structural elements and provided service systems [[1], [2], [3], [4], [5], [6],^18^,^24^,^27^,^33^,^38^,^39^]. The content of the present paper is limited to the methodological details of building sustainability assessment indicator scoring, which is reproducible for any city. The remainder of the RSAM methodology along with indicator weights, which are more specific to a study area, are discussed in the associated article in full details [17].

In this study, stakeholders can be defined as people who have interests in, can influence, or be influenced by a company or organization. In the present study, the term “stakeholder” refers collectively to a multitude of stakeholder groups such as the general public, academicians, and construction workers. Householders (also called as residents or occupants in this study) are one of the stakeholder groups and can play a pivotal role in the sustainability assessment of the building as they either occupy or are in charge of the house or the apartment.

All the indicators are scored in quantitative terms using a Ten Points Scaling (TPS) [17]. The primary goal of assigning points was to reveal implementations that were better or superior to the average or common applications within the context of a selected city. Most indicators require no adjustments for the studied urban context and can be directly applied to any building in any city, but some require city-specific information to use (e.g. ENV 2.4, ECO 1.1, ENV 4.1, and S&F 5.1). Each indicator rating is designed to support a rapid assessment based on resident opinion, and they should not be complex or very technical. Since this is the first evaluation in the literature, which is largely resident opinion-based, the indicator assessment methodologies and their suggested scores were developed from the ground up during targeted stakeholder workshops [17], and they are not obtained from an earlier method.

## ENV: Environmental factor

Construction activities, as well as operation of buildings, have potential to damage and/or create harmful impacts on the environment, namely air, soil, water, and biodiversity if they are not carefully designed and constructed [9,10,37]. Even if the potential negative impacts of construction activities along with the emissions and waste discharges during the building operation time are controlled and limited, a certain amount of (generally acceptable) adverse environmental impact may still occur due to emissions, pollutions, and physical design and layout of buildings that have already occurred and implemented. RSAM covers the main environmental indicators of existing buildings in four titles as follows: i) energy; ii) soil use and biodiversity; iii) materials used and solid waste; and iv) water.

### ENV1: Energy

Energy category is an important parameter in the building sustainability assessment. Based on the recent regional studies and audit reports, energy consumption per area and insulation characteristics are identified as the most significant indicators among several energy-related parameters to characterize the energy performance of residential buildings in Kazakhstan [19]. Consequently, energy consumption, energy production, and insulation are the three major indicators used to evaluate the level of energy efficiency and sustainability of the building in this category.

#### ENV1.1: Energy consumption

This indicator is commonly used in most of the assessment methods and usually assigned many points [30]. The knowledge of how to quantify the energy use in buildings helps to set better energy efficiency goals. It is selected to improve the inherent energy performance of the building, encourage the reduction of carbon emissions, and support efficient management throughout the operational phase of the building's life cycle. ENV1 is designed to measure the energy consumption levels and reward any significant attempt to reduce these levels in buildings. Householder opinion is rated using 5 levels of satisfaction (1: “not happy”, 2: “marginally happy”, 3: “happy”, 4: “very happy”, and 5: “extremely happy”) with the building's energy consumption, and the scores are assigned accordingly (0 points (in 10-Points Scale (TPS)) for being “not happy” and more for higher levels of satisfaction, respectively).

#### ENV1.2: Insulation

The energy performance of the building can be evaluated considering the heat loss due to layers of insulation with the available information on materials used. R-values (the insulation's resistance to heat flow) are commonly used in describing the performance of the insulation, and good insulation is a typical insulation system with suggested R-value for a specific climate region [12]. Due to the high level of technicality in the calculation of the R-value as well as unavailability of data on the insulation materials of certain buildings, it cannot be employed in the presented method. Instead, this method proposes to evaluate the current insulation performance of the building by comparing it to an “optimal” case for insulation only. If householder agrees with the performance of their building's insulation to be “ideal”, it will be given the score of 5; otherwise, the scores are assigned according to Table 1.

**Table 1:** Assessment summary for “insulation” sub-indicator.

Opinion (Evaluating the current performance of your building insulation by comparing to an “ideal” case for insulation only)	Points
Extremely poor	0
Poor	2
Optimal	5
Better than optimal	7
Excellent	10

#### ENV1.3: Energy production

Renewable energy technologies in residential buildings have become more effectively used during the last decade [14]. The availability of such technologies (e.g. solar panels, wind turbines) is considered to be an important part of the buildings’ energy efficiency, even of the residential buildings, and increase the sustainability level significantly [25]. ENV1.3 sub-indicator measures the performance of all present renewable energy systems installed/used by the building. If the building has not any relevant application, 0 points is given; while 2, 4, 6, 8 and 10 points are given for 10%, 30%, 50%, 70% and 90% coverage of the annual energy consumption, respectively.

### ENV2: Soil use and biodiversity

“Soil use and biodiversity” is the second indicator of the “environmental” factor. It is characterized by six quantifiable sub-indicators as follows.

#### ENV2.1: Layout improvement and optimization

Sustainable buildings start with proper layout selection and/or improvements which offer significant sustainability benefits. The location of the building affects a wide range of environmental factors as well as the factors including a selection of a proper site, site improvements, security, accessibility, and impacts on the local ecosystem. Moreover, it evaluates if the project is located along with the existing and future watershed planning projects that are already in place. Eight questions were designed accordingly (e.g. “does your building reduce, control, or treat surface runoff through effective stormwater practices?”), and each positive answer collects 1.25 point in the assessment (Table 2).

**Table 2:** Checklist for “layout improvement and optimization” sub-indicator.

Sub-indicator	Checklist
Layout improvement and optimization	Does your building retrofit existing buildings?Does your building minimize the creation of new impervious surfaces (parking, building layout, travel lanes, etc.)?Does your building consider efficient watershed plans?Did your building consider energy implications and carbon emissions in site selection?Did your building apply best practices for erosion control?Does your building use native plants and remove any invasive species?Does your building minimize habitat disturbance?Does your building reduce, control, or treat surface runoff through effective stormwater practices?

#### ENV2.1: Soil sealing

Soil sealing is a permanent cover of soil by completely or partially impermeable artificial materials (e.g. asphalt, concrete), buildings and infrastructure [31]. Urban areas are characterized by a high degree of soil sealing and by a high concentration of urban infrastructure, which includes all forms of pavements and buildings. This increases the amount of runoff water as well as average temperature impacting microclimate characteristics of the area. Furthermore, it has negative effects on biodiversity. Due to its negative effects on the ecosystem, it is crucial to evaluate the sustainability level of structures considering soil sealing. In order to assess the building's performance in terms of this sub-indicator, eight assessment items were identified. Any positive response, activity, or improvement on these selected items are given 1 or 2 points. “Securing of flora and fauna habitat” and “climate adaptation improvements” (e.g. decrease in heat emission, carbon binding) worth 2 points, while the other items (e.g. “control of surface water runoff”, “improvement of private recreational areas” (gardens, courtyards), “improving public green areas” (e.g. parks), “improving recreational areas” (e.g., forests, landscape parks), “protection of agricultural areas”, “protection of ecologically valuable fertile soils”) are awarded 1 point (Table 3).

**Table 3:** Checklist for “soil sealing” sub-indicator.

Indicator	Checklist
Soil Sealing	Securing of flora and fauna habitatsControl of surface water runoffClimate adaptation improvement (e.g. decrease of heat emission, carbon binding)Improvement of private recreational areas (gardens, courtyards)Improving public green areas (e.g. parks)Improving recreational areas (e.g., forests, landscape parks)Protection of agricultural areasProtection of ecologically valuable fertile soils

#### ENV2.3: Reuse of previously built or contaminated areas

Reuse of previously built or contaminated areas can be defined as an extent of utilization of land that is contaminated or was previously occupied [21,28]. This sub-indicator is beneficial to the environment as urban sprawl is restrained and greenfield sites are preserved. Moreover, reusing vacant built sites supports the restoration of former landscapes and may reduce energy and material consumption. In the case of areas with contaminated soil, reuse of the land promotes treatment of contaminated soils [28]. However, it poses a potential threat to the environment as well as human health due to the possible increase in exposure to contamination. Therefore, it is essential to evaluate the site on the reuse of previously built or contaminated areas. A simple approach is suggested to evaluate this sub-indicator: TPS scores are provided by assigning 1 point to 10% of area reuse of previously built or contaminated areas following the decontamination process. For example, if the estimated level of soil decontamination is 40%, the corresponding score is 4 (Table 4).

**Table 4:** Inquired information for ENV2.3 and ENV2.4 sub-indicators.

Reuse of previously built or contaminated areas	Ecological protection of the site
The area of the territory that was previously used (m^2^)The area of the territory that was previously contaminated (m^2^)The area of decontamination, (m^2^)	Selection of the site for ecologic survey by the authoritiesPresence of habitats within 100 m (e.g. woodlands, water sources, wetlands, flower-rich lands)Destruction of mature hedgerow or trees older than 10 yearsPresence of one of the Nature Reserves of the region within 2 km

#### ENV2.4: Ecological protection of the site

This sub-indicator can be defined as an encouragement to protect the biodiversity of the local area. In order to define a building's ecological value, four criteria were chosen. Initial TSP score for ENV2.4 is set to 10 points, and the scores for the selected criteria are subtracted from this initial value. The first criterion is the selection of the construction/building site according to ecological surveys performed by authorities, which identifies the habitats and/or species that exist within the area. If the building was not originally constructed on a site satisfying the requirements of an ecological survey, 2 points are deducted from the initial value of 10 points. Three more points are subtracted from the total value, if there are one or more habitats within 100 m radius of the buildings (e.g. woodlands, water sources, wetlands, flower-rich lands). The existence of mature hedgerow or trees older than 10 years in Nur-Sultan and its surrounding area is very limited, and their protection is a priority. If there were hedgerow or trees on the construction site, which were cut due to the construction activities, 2 points are subtracted from the total score. The last criterion is the proximity of Nature Reserves of the study region: if there is at least one in the vicinity of the building (2 km or closer), the building loses 3 points (Table 4).

#### ENV2.5: Soil rehabilitation

This sub-indicator is essential for preventing soil degradation. Soil is an important component of the environment system. Intensive and increasing use of land due to anthropogenic activities leads to its degradation and pollution, which may result in a partial or complete loss of its productive capacity. Thus, it is crucial to rehabilitate soil by a number of different measures, including vegetation planting. TPS assessment is based on rating (out of 10) on a proportion of rehabilitated land to effective land multiplied by 10.

#### ENV2.6: Use of native plants

Promotion of the use of native flora is advantageous in many aspects. Some benefits include cost-effectiveness, reduction in energy consumption, positive impact on biodiversity, increased tolerance of the plants to local conditions (climate, soil conditions, etc.), fitness for the local landscape, and contribution to plant reproduction. Similar to ENV2.5, the assessment of “use of native plants” sub-indicator is directly related to the ratio of surface area covered by native plants to the vegetative area (“Is there any imported (not native) plant species on the building site? If yes, provide an approximate number of plants and area coverage”).

### ENV3: Materials and solid waste

Materials used during the construction period and solid waste management practices during the operational period of the assessed building constitute the third indicator of “environmental” factor.

#### ENV3.1: Reused products and recycled materials

The use of recycled and reused materials offers sustainability in the construction sector by diverting waste from landfills and reducing the use of raw materials and energy, air and water pollution, as well as overall environmental impact [13]. This sub-indicator is evaluated by identifying the percentage of reused and recycled materials in the total amount of material used (10 points for ≥15%, 8 points for ≥12%, 6 points for ≥8%, 2 points for ≥3%, and 0 points for ≤3%).

#### ENV3.2: Waste separation and storage

This sub-indicator measures the sustainability level of solid waste management practice in existing buildings. Waste separation and storage is a common practice in developed countries. In the method, it is evaluated by assessing the following three parameters. The first parameter checks the presence of space in the building or adjacent to the building suitable for storage of wastes/recyclables (2 points). The second parameter is the availability of separate containers for different categories of household waste (3 points). And the last one evaluates the condition of the containers (5 points for Excellent, 4 points for Good, 3 points for Moderate, 1 point for Poor) [29] (Table 5).

**Table 5:** Checklist for “waste separation and storage” sub-indicator.

Subcategory/criteria	Description	Points
Presence of suitable space in the building or adjacent to the building for storage of wastes/recyclables	Yes	2
	No	0
Availability of separate containers for different categories of generated solid waste	Yes	3
	No	0
Level of containers’ condition. Ex: closed-open, readiness for hauling, roofing, etc.	Excellent	5
	Good	4
	Moderate	3
	Poor	1

### ENV4: Water and wastewater

Water and wastewater management strategies on the building scale contribute to the sustainability level of the structure. “Water and wastewater” indicator is quantified using four sub-indicators which are the most relevant ones according to the householder opinion-based sustainability assessment.

#### ENV4.1: Water consumption

Management of potable water consumption in buildings is one of the most important sustainability factors, especially in the countries with water scarcity and issues with the quality of drinking water due to poor water supply systems [16]. In general, on the building scale, the sustainability performance of water systems is mainly dependent on interior design decisions (e.g. use of water-conserving fixtures and water-efficient appliances, etc.) and user experience and behaviour (e.g. daily practices, aesthetics and hygiene requirements, reliability/durability of the provided system, etc.). Consequently, its quantification may require detailed analyses in addition to site investigations and surveys. The approach utilized in the discussed method offers a relatively easy and convenient way of the assessment based on employing water consumption values. It compares the average water consumption per person of the building to the average water consumption in the region. If the value is more significant or equal to the national average, 0 points are given. Every cubic meter decrease in personal water consumption is awarded two additional points. For example, if the buildings’ water consumption per person is around 3 m^3^ less than the national average, 6 points are awarded (Table 6).

**Table 6:** Assessment Summary for “water consumption” sub-indicator.

Water consumption (per person per year) (Rounded up values)	≥national water consumption per person (m^3^)	1 m^3^ less	2 m^3^ less	3 m^3^ less	4 m^3^ less	5 m^3^ (or more) less
Assigned points	0	2	4	6	8	10

#### ENV4.2: Recycling and reusing of grey water (excluding rain)

In terms of sustainability, there is a great potential in the reuse of greywater, because it might be utilized for many applications such as toilet flushing and other non-sanitary uses. Recycling and reusing greywater will reduce the demand for potable water as well as the amount of wastewater requiring treatment [34]. Regardless of the technique used to recycle/recover grey water, 1 point is given for every 10% of water recycled or reused of the amount of water consumed. For example, if the building consumes 12 cubic meters per person annually and reuses 20% of the treated greywater, the building earns 2 points.

#### ENV4.3: Rain and stormwater collection and use

Rain and stormwater collection and use is an ancient practice as an additional source of water and is a good sustainability practice in water consumption in houses. The assessment of this sub-indicator is similar to ENV1.3 and is based on the ratio of the amount of water harvested to the amount of water consumed (3 points for 0-25%, 5 points for 25-50%, and 10 points for >50%).

#### ENV4.4: Separation of black water

Blackwater comes from toilets and has high organic load as well as large numbers of pathogens. Any kind of separation of black water from grey water will lead to improved treatment of greywater. Systems for separation of grey and black water are rare in developing countries; therefore, 10 points are awarded for implementing any form of the separation system, otherwise – zero points.

## ECO: Economic factor

In the existing literature, economy is one of the bottom lines in building sustainability assessment studies and has been assessed by many indicators and sub-indicators [23] focusing mostly on the construction stage. This study, too, supports the idea of economic bottom line theory, but it simplifies the procedure by using only two main indicators: i) the cost of the building including initial and operational costs (ECO1), and ii) how the building supports the local economy (ECO2).

### ECO1: Cost of building

The cost of the existing residential building for stakeholders is assed based on the initial and operational costs.

#### ECO1.1: Initial costs

A sustainable building (e.g. smart, green, people-friendly, etc.) may cost more upfront (initial costs), which generally translates to a higher purchase price for householders, but can provide opportunities for savings over the building's life cycle through lower operational costs [22]. However, it does not mean that more expensive buildings should get higher scores in the assessment: the lower the purchase costs become – the greater the score awarded by the framework. The TPS assessment utilized in this framework is based on a ratio between the purchase cost of 1 m^2^ of the building and an average market cost of 1 m^2^ of residential buildings in the study area. Ten TPS scales are defined based on this ratio and their ranges such that more expensive buildings are rated with lower points (e.g. 10 points for <65%, 9 points for 65-70%, 8 point for 70-75%, 6 points for 80-85%, 5 points for 85%-95%, 4 points for 95-105%, 3 points for 105-115%, 2 points for 115-125%, and 1 point for >125%). The relationship between the cost of the building and the number of points awarded is not linear (Table 7).

**Table 7:** Assessment Summary for “initial costs” sub-indicator.

Points	1	2	3	4	5	6	7	8	9	10
Initial cost ratio	>125%	125%-115%	115%-105%	105%-95%	95%-85%	85%-80%	80%-75%	75%-70%	70%-65%	<65%

#### ECO1.2: Operational costs

From the perspective of householders, the operational cost elements representing the sustainability of the building are identified as utilities, service costs, and operational costs represented through savings. Energy use and water consumption are assessed in ENV1.1 and ENV4.1, respectively. Building service cost is also assessed in a similar way by collecting householder opinion on the total cost of the building compared to the average cost of the city (using the same scale as ENV1.1). ECO1.2 is the average value of these three scores.

### ECO2: Local economy

There is a consensus among sustainability studies that supporting the local economy (i.e. maximizing opportunities for local businesses, labour, and training) promotes viability and provides more sustainable solutions [7,11,26]. In order to identify to what extent the building has been supporting the local economy, “hiring local goods and services” is selected as a sub-indicator.

#### ECO2.1: Hiring local goods and services

This sub-indicator assesses the cost of hiring local goods and services (0.5 x local goods points + 0.5 x local services points) during the operational stage. The estimated per cent of total money spent on local materials and goods in building repairs, maintenance and any other building activities is multiplied by 5 (e.g. for 50% local resource use, 0.5*5=2.5 points). And, the point assignments up to 5 points using the same way on the per cent of total money spent on local services.

## S&F: Social and functional factor

“Social and functional” factor encourages an integrated design process that optimizes the building's performance. This category rewards the implementation of processes and strategies that support positive sustainability outcomes. The category also promotes practices which ensure that the project is used up to its optimum operational potential. It consists of five indicators: S&F1: “user's health and comfort”, S&F2: “passive design”, S&F3: “mobility plan”, S&F4: “space flexibility and adaptability”, and S&F5: “facilities”.

### S&F1: User's health and comfort

#### S&F1.1. Natural ventilation

The general purpose of ventilation in buildings is to provide fresh air. RSAM approach encourages the use of natural ventilation over mechanical alternatives, and it is based on quantifying the usage of natural ventilation vs mechanical ventilation during a whole year period. The smaller number of days of HVAC or any other type of mechanical ventilation usage is rewarded with more points in the assessment (e.g. 10 points for a maximum of 20 days of usage versus only 1 point for more than 310 days of usage) (Table 8).

**Table 8:** Assessment summary for “natural ventilation” sub-indicator.

Rating Value	1	2	3	4	5	6	7	8	9	10
Number of days with mechanical ventilation	311-365 days	275-310 days	238-274 days	202-237 days	165-201 days	129-164 days	92-128 days	56-91 days	20-55 days	0-19 days

#### S&F1.2: Toxicity of finishing materials

It is not easy to evaluate toxicity levels of the materials in the existing building since they have already been embodied in the structural components. RSAM suggests evaluating this parameter based on the responses or awareness of residents about existing material standards and green certifications of their building. The rating values are 0 for no certified materials, 2 for satisfying some standards and having some certification, 4 for all materials satisfying the standards, 7 for having some green certification, and 10 for having all necessary green certification (Table 9).

**Table 9:** Assessment Summary for “toxicity of finishing materials” indicator

Rating Value	0	2	4	7	10
Level of material certification	No certified materials were used	Standard certifications are not provided	All materials satisfy the standards	Some form of green certification was acquired	All necessary green certification was acquired

#### S&F1.3: Thermal comfort

Thermal comfort may be experienced differently based on personal preferences. As a result, it is considered as a subjective parameter, which depends on the individual experience. RSAM considers this situation in rating assignments. Rating values are assigned in accordance with the sensation definitions from Predicted Mean Vote (PMV) index which refers to a thermal scale ranging from “cold” (-3) to “hot” (+3) originally developed by Fanger and later adopted as an ISO standard [36]. PMV value is calculated by subtracting experienced average temperature of the building from a preferred average temperature for the thermal comfort. For example, Nur-Sultan and its surrounding area are in a subtype of humid continental climate with extremely continental weather [20] and the preferred average thermal comfort temperature values of the interviewed householders in this study are 16 ±3°C and 20 ±2°C for summer and winter periods, respectively. Then, the maximum point (10) is given, if the perceived value is neutral (0), and rating points are deducted for increasing deviations (e.g. cold and hot sensations get the minimum points in the rating scale) (Table 10).

**Table 10:** Assessment Summary for “thermal comfort” indicator.

Value	Sensation	Rating Value
-3	Cold	4
-2	Cool	6
-1	Slightly cool	8
0	Neutral	10
1	Slightly warm	8
2	Warm	6
3	Hot	4

#### S&F1.4: Visual comfort

Maintaining visual comfort means ensuring that people have a sufficient amount of light for their activities, the light has the right quality and balance, and people experience pleasant views. Good lighting helps to create a happy and productive environment, but natural light surpasses electric lighting without consuming energy. Use of natural light is evaluated in S&F1.7, so this indicator is based only on the user experiences reported by the residents. The maximum score of 10 points is given if the residents report that they are very comfortable in all the places in the building including private (their apartments) and common areas (e.g. building entrance, corridors, etc.). The rating in this category is reduced according to the level of negative experiences, and none of the points is awarded if they are not comfortable anywhere in the building (Table 11).

**Table 11:** Assessment Summary for “visual comfort” indicator.

Rating Value	0	2	4	8	10
Residents’ opinion	Not comfortable in any place in the building	Several complaints about private and common places in the building	Comfortable in flats but some complaints on common areas	Good level of illumination in all the parts of the building	Very good level of illumination in all the parts of the building

#### S&F1.5: Acoustic comfort

Acoustic comfort can make a significant contribution to the sustainability levels in terms of functionality of all building types, especially in residential buildings, since it drastically affects the life quality of residents in multiple ways. Four criteria (applications) are identified to rate the quality of acoustic comfort in residential buildings: i) exterior acoustic isolation (from the outdoor environment) (4 points), ii) interior acoustic isolation (from common areas and other flats) (3 points), iii) interior soundproofing from different spaces within the flat (other rooms) (2 points), and iv) any other applications in the building (e.g. silent appliances) (1 point). In case of having proper applications in each application categories, householders’ opinions are also collected in three assessment options as “very good”, “satisfactory” and “not satisfactory” [8] (Table 12).

**Table 12:** Assessment Summary for “acoustic comfort” sub-indicator.

Indicators	Score factor	Score
Soundproofing with outdoors	Very good	4
	Satisfactory	2
	Not satisfactory	1
Soundproofing with indoors with other flats	Very good	3
	Satisfactory	1.5
	Not satisfactory	0.5
Soundproofing with indoors within the flat (other rooms)	Very good	2
	Satisfactory	1
	Not satisfactory	0.5
Any appliance working in the rooms (washing machine, etc.)	No appliance	1
	One appliance	1
	More than two appliances	0.5

#### S&F1.6: Indoor air quality (IAQ)

Indoor air quality (IAQ) is the air quality within the building, which has significant potential to impact the health of householders. Four main measures are identified to assess the sustainability of buildings in terms of air quality: i) householder responses or complaints on their health conditions, ii) number of occupants per square meter, iii) perceived humidity in the building, and iv) facility ventilation system. The information based on the householders' reports in terms of experienced symptoms of sick building syndrome is important, since it might be a direct indication of toxic or unhealthy material utilization and/or other conditional factors (e.g. radon gas exposure) in the building [32]. If no report or complaint is received, the building scores 2.5 points. The points are reduced to 1.5 and 0.5 in cases of receiving reports or complaints from a single person and several people, respectively. We applied the same scoring approach to other meters as well (e.g. 2.5, 1.5 and 0.5 points for<4, 4<N<6, and >6 occupants per square meter, respectively) (Table 13).

**Table 13:** Assessment Summary for “IAQ” sub-indicator.

Indicators	Factor	Score
Householder responses (complaints) on their health conditions	No report on the presence of illness symptoms	2.5
	Only one report on the presence of symptoms	1.5
	Several reports on the presence of symptoms	0.5
Number of people /m^2^	<4	2.5
	4<N<6	1.5
	>6	0.5
Humidity in the building	Comfortable all the time	2.5
	Comfortable most of the time	1.5
	Sometimes uncomfortable	0.5
Facility ventilation system	Excellent air mixing	2.5
	Acceptable air mixing	1.5
	Poor air mixing – the presence of the dead spots with poor air movement	0.5

#### S&F1.7: Natural light

The use of natural light is a significant parameter in the building's functionality category. The rating of this sub-indicator was performed based on the ratio of window-to-floor areas of the building. Firstly, the building's total area of the floor and the total area of all windows exposed to outdoors are calculated. Then, their ratio (window/floor) is used for assigning points in this sub-indicator. Any value higher than 15% is awarded 10 points, and the awarded points are reduced proportionally to the percentage values (Table 14).

**Table 14:** Assessment summary for “natural light” sub-indicator.

Points	0	3	4	7	9	10
Window-to-floor area ratio	No availability of natural light	Less than 4%	Between 4 and 5%	Between 5 and 10 %	Between 10 and 15 %	More than 15%

### S&F2: Passive design

#### S&F2.1: Layout and orientation

The land use percentage of building along with the available green area, bicycle parking, and general accessibility to the building is considered as the main factor in “layout and orientation” sub-indicator. It promotes the availability of non-building and free of soil sealing spaces in the residential site (e.g. green area and outside parking zones). There is a substantial variety (10-90%) among cities in the sustainability threshold levels for the available free-of-soil-sealing area [15]. In Nur-Sultan, 60% or more for a free-of-soil-sealing portion of the site in a residential site is perceived as sustainable; thus, this value is awarded 10 points, and the amount of given points is reduced as the free-of-soil-sealing portion of the land reduces (Table 15).

**Table 15:** Assessment summary for “layout and orientation” sub-indicator.

Points	10	8	6	4	2	0
% of building occupation	Less than 40%	Between 40 and 60 %	Between 60 and 80 %	Between 80 and 95 %	Between 95 and 99 %	100%

#### S&F2.2: Passive systems

Passive design strategies provide more environmentally friendly solutions than purchased energy such as electricity or natural gas, but they are more effective in temperate climate conditions. The most reasonable passive strategies for the case of Nur-Sultan are listed and rated as follows: use of any means of solar energy (5 points), availability of a passive ventilation system (different than regular windows) (3 points), and other passive methods or technologies (e.g. humidity control, odour control, air-tight construction, super insulation, good orientation of the building, self-shading design, etc.) (2 points).

### S&F3: Mobility plan

#### S&F3.1: Occupant safety

Some parameters related to the occupant safety and health have already been assessed in other sub-indicators (e.g. Indoor Air Quality). This sub-indicator measures the perceived overall safety of occupants. After giving relevant information about what is occupant safety (e.g. it is the potential for indoor air quality problems, occupational illnesses and injuries, exposure to hazardous materials, accidental falls, slips, trips, and other injury possibilities, nuisance control including mental as well as physical health), residents’ opinions are collected in the range of unacceptable (1) to outstanding (10) (Table 16).

**Table 16:** Assessment summary table for the sub-indicators of “mobility plan” indicator.

Points	10	8	5	3	1
Occupant safety	Outstanding	Very good	Average	Poor	Unacceptable
General accessibility	0-2 min/0-20% (Outstanding)	2-5 min/21-50% (Excellent)	5-8 min/51-80% (Average)	8-9 min/81-90% (Poor)	**>**10 min/>91% (Unacceptable)

#### S&F3.2: Accessibility (general accessibility and accessibility for disabled people)

The aim of improving accessibility of the building is to enable people with disabilities to live independently and is assessed by comparing ease and comfort of general human movement through the spaces inside the building to those of disabled people. The evaluation rating is the per cent difference in travel time from a starting location (e.g. apartment room) to a target location (e.g. residential site exit) between the general public and people with disabilities. The rating ranges from less than 20% difference, outstanding (10 points), and more than 91%, unacceptable (1 point) (Table 16).

### S&F4: Space flexibility and adaptability

The space flexibility and adaptability are measured using two sub-indicators as follows.

#### S&F4.1: Availability and accessibility to social areas

This sub-indicator uses a similar method to S&F3.1 and measures the perceived overall occupants’ satisfaction with the availability of social areas and their accessibility. Ratings are given in the range from unacceptable (1) to outstanding (10).

#### S&F4.2: Space optimization

“Space optimization” sub-indicator aims to measure utilization efficiency of residential buildings, and the following procedure is used to assess it. First, a ratio of “usable area of building” to “gross area of building” is calculated. The ratio value of a previously defined level (0.80 for this method) is perceived as an excellent space optimization performance for a given urban context. Thus, the highest number of points (10) is awarded, if the value is achieved or exceeded, and 1 point is deducted for each 5 per cent decrease in the ratio.

### S&F5: Facilities

#### S&F5.1: Accessibility to public transport

The proximity of the building to public transport stations is identified as an important sustainability factor for householders [35]. The method assesses this sub-indicator by considering not only the distance to the station but also the number of bus routes passing through this station. Threshold values were assigned by collecting stakeholders’ opinions for perceived excellent distance and a number of routes (5 minutes and three bus routes for the study city, respectively). Then the sub-indicator rating is calculated by referring these thresholds along with the average walking time from the building to the bus stop and the number of bus routes passing through that bus stop (Table 17).

**Table 17:** Rating summary table for the sub-indicators of “facilities” indicator.

Sub-indicator	Rating method
Accessibility to public transport	In assessing accessibility to public transport, not only the distance to the nearest bus stop but also the number of bus routes passing through this bus stop are important. Excellent walking time and the number of buses are taken like 5 minutes and three bus routes, respectively. According to these values and assuming equal importance, the following equation is used:$Rating=5*\frac{5\;}{x}+5*\frac{y}{3}$Where x is time spent to walk from the building to the bus stop, and y is a number of bus routes passing through that bus stop. If the calculated value exceeds 10, the value of 10 is used.
Local amenities	The list of amenities with weights in brackets are as follows: schools (20), hospitals (1), supermarkets/stores (25), banks/ATM (3), public transport (20), parks (8), and shopping malls (3). This information was used to build the equation below:$Distance=\;\sum f_{i}d_{i}/7$Where $f_{i}$- weight of the amenity, and $d_{i}$- distance from the amenity to the building.Distance Rating10 points ≤1 km8 points 2 km5 points 3 km3 points 4 km1 point ≥5.5 km
Low impact mobility	Assessment of low impact mobility requires the identification of how many times per month householders are attending local amenities by walking/cycling (Mode 1) and by driving a car (Mode 2). The ratio of Mode 1 to Mode 2 is rated as follows:Mode 1/Mode 2 Rating10 points >0.98 points 0.75 points 0.43 points 0.21 point <0.1
Building monitoring	Services provided after delivery for use.1 point: Fire and Security Monitoring1 point: Fire Alarm Monitoring1 point: Intrusion Alarm Monitoring1 point: Elevator Phone Monitoring1 point: Building Automation Monitoring1 point: Lighting Monitoring1 point: HVAC Monitoring1 point: Critical Point Monitoring1 point: Sanitation Monitoring1 point: Air Quality1 point: Water Hygiene

#### S&F5.2: Local amenities

Proximity to local amenities improves the residential building's sustainability and potentially decreases environmental impacts (e.g. reducing traffic emissions). However, the importance of distance is different for different amenities. For instance, some amenities such as grocery stores are visited every day; thus, it is more important to live closer to grocery stores than a hospital. Therefore, this method uses amenity factors to calculate a weighted rating value for this sub-indicator. Seven main amenities are listed, and their scores are assigned as follows: schools (20), hospitals (1), supermarkets/stores (25), banks/ATMs (3), public transport (20), parks (8), and city malls (3). Then an average distance value is calculated using the amenity factors and distances from the building to the corresponding amenities. TSP scores were assigned to the average distance values between ≤1 km and ≥5.5 km (Table 17).

#### S&F5.3: Low impact mobility (walking/cycling)

Assessment of low impact mobility requires the identification of how many times per month householders are attending local amenities by walking/cycling and by driving a car. The ratio between these two modes of mobility is calculated and rated (Table 17).

#### S&F5.4: Building management and availability of services

Services considered in the evaluation of this sub-indicator consist of “fire and security monitoring”, “fire alarm”, “intrusion alarm”, “elevator phone”, “building automation”, “lighting”, “HVAC”, and “critical point” (including sanitation, air quality, and water hygiene) monitoring. All available services are awarded 1 point summing up to a total of 10 points (Table 17).

• *Supporting data.xls*
• The method presented provides details for a residents’ opinions-based sustainability assessment procedure: Rapid Sustainability Assessment Method (RSAM), specially designed for existing residential buildings. It was tailored to the needs and characteristics of the urban structures in Kazakhstan. It aims to provide reliable data to construct a bridge between the identified shortcomings of existing buildings and future sustainability requirements. Though the method was developed for existing residential buildings specifically, its flexibility allows for the inclusion of data obtained from various stakeholders (e.g. building management) as well as making adjustments to assess non-residential buildings or apply to different city contexts. Assessments of 12 different building complexes in Nur-Sultan have also been performed to test the performance of the model and then presented in the accompanying original research article.
• In Kazakhstan, the progress towards sustainable construction is thwarted mainly by outdated construction standards and regulations, a low engagement of the construction industry in green projects, and a lack of general awareness or academic research in the field of sustainability. The context in which RSAM was developed renders it potentially suitable for developing countries experiencing similar constraints: the method is relatively quick, inexpensive, has the structure which is easily adaptable to a different set of components and their weightings, and does not require in-depth knowledge on sustainability from the respondents (in the current case, householders). However, the framework's flexibility allows modifications on the structure by adding or removing different indicators and on the weighting system by assigning weights appropriate for any region or context, not only developing countries or residential buildings.
• The limitations to the developed framework include the following: (1) limited sustainability research in Kazakhstan; (2) the relative newness of sustainability domain to the public and need in some on-site education on the subject prior to the survey; (3) the inherent subjectivity in the assessment of certain sub-indicators (e.g. perceived average temperature); (4) difficulties in acquiring accurate data in older buildings (such as energy or water consumption) due to the absence of measuring devices (meters) or unavailability of records. The future work involves the improvement of the developed method by countering identified limitations as well as an expansion of the framework's scope by adding more building types which can be assessed by the framework.

## Appendix Supplementary materials

Supplementary material and/or Additional information: Image, application 1
